# Association of Altered V1-M2 Neuronal Activation with Balance and Coordination Deficits in a Corneal Alkali Burn Mouse Model of Visual Impairment

**DOI:** 10.3390/brainsci16080774

**Published:** 2026-07-23

**Authors:** Yunan Zhou, Xiaoming Shi, Ruilan Dai, Mingxuan Gao, Yingfang Ao, Guogang Xing, Jin Cheng, Mingxin Ao

**Affiliations:** 1 Tianjin Key Laboratory of Exercise Physiology and Sports Medicine, Institute of Sport, Exercise & Health, Tianjin University of Sport, Tianjin 300381, China; 2 Beijing Key Laboratory of Sports Injuries, Beijing 100191, China; 3 Department of Sports Medicine, Peking University Third Hospital, Institute of Sports Medicine of Peking University, Beijing 100191, China; 4 Department of Neurobiology, School of Basic Medical Sciences, Peking University Health Science Center and Neuroscience Research Institute, Peking University, Beijing 100191, China; 5 Department of Ophthalmology, Peking University Third Hospital, Beijing 100191, China

**Keywords:** visual impairment, balance and coordination function, sensorimotor integration, primary visual cortex, secondary motor cortex

## Abstract

**Highlights:**

**What are the main findings?**

- Visual impairment in mice caused by corneal alkali burns can lead to decreased balance and coordination.
- Visual impairment reduces balance and coordination, associated with attenuated activation in the V1-M2 neural circuit.
- V1-M2 circuit neuronal activation is reduced in visually impaired mice during visually guided motor tasks.

**What are the implications of the main findings?**

- The present findings support the V1-M2 circuit as a candidate pathway underlying visual impairment-related visuomotor integration changes and warrant further investigation via causal manipulation approaches.
- Balance and coordination measures might be more sensitive indicators of visual impairment-related motor dysfunction in mice.

**Abstract:**

Background: Visual impairment frequently impairs balance and motor coordination, yet the underlying neural circuit mechanisms remain poorly understood and require further investigation. In this study, we established a mouse model of visual impairment to assess the effects of defective vision on balance and coordination and further elucidated the functional role of the V1-M2 neural circuit in this process. Methods: A mouse model of corneal alkali burn was generated. Corneal morphology was observed, visual function was detected, and balance as well as motor coordination were evaluated. Neuronal activation in the V1 and M2 brain regions was quantified via c-Fos immunostaining. Viral tracing was performed to map the V1-M2 neural pathway, and chemogenetic manipulation was applied to modulate the V1-M2 circuit. Results: Mice subjected to corneal alkali burn exhibited abnormal corneal morphology and impaired visual function, accompanied by significant deficits in balance and motor coordination. The number of c-Fos^+^ neurons was markedly reduced in both the V1 and M2 regions. The direct V1-M2 neural circuit was anatomically verified. Chemogenetic activation of the V1-M2 circuit elevated c-Fos expression in M2 and rescued impaired motor performance. Conclusions: Visual impairment disrupts balance and motor coordination in mice. The V1 and M2 cortical areas mediate this behavioral dysfunction, likely due to attenuated activation of the V1-M2 neural circuit. These findings identify a promising neural circuit for dissecting the fundamental mechanisms underlying visuomotor integration.

## 1. Introduction

Motor balance and coordination are integrative abilities that enable the body to perform diverse movements smoothly and accurately [1]. The execution of this function relies primarily on the input, integration, and processing of sensory information within the central nervous system [2]. Visual feedback plays a critical role in motor execution by providing real-time guidance to facilitate the adjustment and optimization of movement trajectories. Therefore, observing the effects of visual impairment on motor function and the concurrent alterations in brain function lays an important foundation for further exploring the neural mechanisms of visuomotor integration.

The neural pathways underlying visual modulation of motor performance involve complex synergistic interactions across multiple brain regions [3]. Visual signals are transmitted from the retina through the optic nerve to the brain, where they undergo complex processing to subserve visuomotor integration [4]. The secondary motor cortex (M2) is involved in the selection of visuospatial information and the modulation of adaptive movements [5]. Previous studies have documented direct monosynaptic anatomical connections between V1 and M2 [6]. We hypothesize that the circuit formed by these two regions may participate in the regulation of motor output by visual signals.

The mouse models of visual impairment are essential for investigating how aberrant visual input leads to deficits in motor balance and coordination. Currently available mouse models of visual dysfunction differ substantially in injury site, onset timing, and severity, each suited to address distinct clinical scenarios. Inherited retinal degeneration models (e.g., rd1, rd10 mice) feature severe progressive loss of retinal function and congenital onset, accompanied by long-term neural remodeling of the visual pathway during development [7,8]. Form-deprivation amblyopia models induce developmental functional inhibition of the visual cortex in addition to peripheral image blurring, introducing intrinsic abnormalities in central visual circuits as a confounding factor [9]. Traumatic optic nerve injury models represent acquired visual impairment, but typically cause severe damage to visual pathway conduction function [10]. The present study aims to investigate acquired mild-to-moderate visual dysfunction occurring in the fully mature normal visual system, as well as its associated motor deficits and cerebral functional changes, while excluding confounding factors of intrinsic optic nerve or visual cortex dysfunction. To this end, we selected corneal opacity induced by corneal alkali burn as the experimental model.

## 2. Materials and Methods

### 2.1. Ethics

All experimental procedures were performed in strict accordance with the guidelines of the Care and Use of Laboratory Animals. The study protocol was reviewed and approved by the Animal Welfare and Ethics Committee of Peking University Health Science Center (Approval No. LA2020353). The study was conducted in compliance with the 3Rs principles for animal research.

### 2.2. Selection and Description of Animals

Male C57BL/6 mice (8 weeks old, weighing 20 ± 1 g) were purchased from the Department of Laboratory Animal Science in Peking University Health Science Center. Twenty mice were used for visual disorder model establishment, visual function assessment, motor function testing, and c-Fos protein detection. Another ten mice were allocated for quantitative analysis via viral tracing. An additional twenty mice were utilized for chemogenetic manipulation of neural circuits.

### 2.3. Technical Information

#### 2.3.1. Establishment of Visual-Impairment Mouse Model

Twenty mice were randomly allocated to either the alkali burn group (10 mice in the group) or the control group (10 mice in the group) using a random number table. Visual impairment was induced via corneal alkali burn. A single-layer circular filter paper disc (5 mm diameter) was immersed in 0.5 mmol/L NaOH solution for 20 s, then briefly blotted on dry filter paper for 1 s to remove excess fluid. Under a surgical microscope, the disc was placed on the cornea of both eyes and gently pressed to ensure adequate contact with the cornea for 10 s. The cornea was immediately irrigated with physiological saline for 1 min [11,12]. Thirty minutes prior to surgery, mice were administered intraperitoneal meloxicam (5 mg/kg) for analgesia. Sodium pentobarbital was used for intraoperative anesthesia to standardize exposure duration and minimize confounding factors. After surgery, ciprofloxacin eye drops and diclofenac eye drops were topically applied once daily to suppress inflammation and relieve ocular pain. On postoperative day 14, mice were anesthetized via inhalation of 3% isoflurane, gently restrained with a dedicated head holder, and briefly examined under a slit-lamp biomicroscope to assess corneal transparency (Model BQ-900, Haag-Streit, Köniz, Switzerland). Corneal opacity was graded according to the Roper-Hall classification: Grade 0, clear cornea; Grade 1, slight corneal haze with easily visible iris and pupil details; Grade 2, moderate corneal haze with visible iris and pupils but obscured iris details; Grade 3, severe corneal haze with barely visible pupil; Grade 4, opaque cornea with invisible pupil. Only mice achieving grade 2 opacity on day 14 post-burn were included in the alkali burn group [13]. Control mice received identical topical treatment under anesthesia, with filter paper discs soaked in physiological saline applied to the corneas for 10 s.

##### Observation on Corneal Clarity

Anterior segment photographs were captured under diffuse and slit illumination with a slit lamp biomicroscope (Haag-Streit, Switzerland; Model: BQ-900).

##### Measurement of Corneal Thickness

Central corneal thickness (CCT) was measured using an optical coherence tomography angiography (OCTA) system (Model RTVueXR, Optovue, Inc., Fremont, CA, USA). Anterior segment optical coherence tomography (AS-OCT) images were acquired in single-line and multi-line scan modes, with CCT measured by the built-in caliper tool.

##### Histopathological Observation of the Cornea

The entire eyeball was excised and fixed in a fixative solution (Feijing Biotechnology Co., Ltd., Fuzhou, China) for fixation. After paraffin embedding, sections were cut at a thickness of 7 μm using a cryostat (Leica, Nussloch, Germany, model: CM1860). The sections were dewaxed with xylene and hydrated with a gradient of ethanol-water. The sample was stained with hematoxylin-eosin staining reagent (Beyotime Biotech Inc., Shanghai, China), sealed with neutral gum, and images were captured using an optical microscope with a 20× objective lens.

##### Flash Visual Evoked Potential Recording for Visual Pathway Function Assessment

Flash visual evoked potentials (fVEP) were assessed using a visual electrophysiology instrument (OPTOPROBE, Pontypridd, UK, model: OPTO-ERG). Mice were dark-adapted for 12 h and then subjected to electrophysiological examination under dim red light. Animals were anesthetized with sodium pentobarbital (80 mg/kg), and the pupils were dilated with compound tropicamide eye drops. The body temperature was maintained with a thermostatic blanket throughout the experiment. Electrodes were placed subcutaneously as follows: the recording electrode was positioned over the occipital cortex, the reference electrode on the cheek, and the ground electrode at the base of the tail. In a dark room, high-intensity brief white light flashes (600 cd/m^2^, 5 ms) were delivered 20 cm from the tested eye at a frequency of 1.3 Hz. Each sweep was acquired over a 250 ms time window. Signals were amplified 4000-fold and band-pass filtered at 1–100 Hz. A total of 65 repeated stimuli were delivered for each measurement, and analyses were performed on the averaged waveforms after signal superposition. During recordings, the placement and contact quality of the ground electrode were strictly controlled, and consistent anesthetic depth, body temperature and electrode positions were maintained in mice throughout data acquisition. The amplitudes and latencies of the N1, P1, N2, and P2 waves were analyzed bilaterally for each mouse, with the P2 wave as the primary outcome measure.

##### Optomotor Response Assay for Visual Acuity Measurement

The optomotor response (OMR) was tested according to the standard protocol established by Prusky et al. (2004) for mice [14]. Visual function was assessed using a virtual optomotor tracking system (OptoMotry system; Cerebral Mechanics, Lethbridge, AB, Canada). The mice were exposed to moving vertical sine-wave gratings (speed: 12°/s, contrast: 100%). Spatial frequencies were increased in a staircase paradigm from 0.042 to 0.6 cycles per degree (c/d). An overhead camera tracked the mouse’s head movements in real time. Horizontally moving sine-wave gratings were presented to both eyes simultaneously. Visual acuity was defined as the highest spatial frequency that elicited consistent head tracking following the direction of grating motion.

#### 2.3.2. Evaluation of Motor Ability

##### Horizontal Ladder Test

The horizontal ladder test apparatus (Nanjing Calvin Biotechnology Co., Ltd., Nanjing, China, model: KW-MGA) consisted of transparent acrylic sidewalls and metal rungs with a diameter of approximately 3 mm. The horizontal ladder consists of acrylic sidewalls (length: 1 m; height: 19 cm) and is fixed 30 cm above the ground, with one end connected to the mouse’s home cage. The width of the baffle is adjusted to 1 cm greater than the mouse’s body width to prevent turning. Metal rungs are irregularly spaced at intervals of 0.5–2.5 cm. One day before the formal test, the mice were given a 5-min training session for adaptation. During the formal test, each mouse traversed the rungs at least 5 times to enter the home cage. Between trials, each mouse rested for 1 min. All tests were video-recorded using a camera (Canon, Tokyo, Japan, model PowerShot V10).

When the mouse traversed 20 consecutive rungs at a constant speed, its hindlimb stepping patterns were observed from the side. From video recordings, we counted the numbers of three types of positive steps (plantar step, toe step, skip step) and three types of negative steps (slip, miss, drag). The definition of hindlimb stepping patterns: Plantar step, the plantar surface of the paw makes initial contact with the rung, with no requirement for full claw grip; Toe step, only the toes come into contact with the rung; Skip step, the hind paw jumps over the rung without any contact; Slip, toes or plantar surface contact the rung but lose balance, causing the foot to drop below the rung plane; Miss, the hind paw passes between two rungs without contact during an attempted gait cycle; Drag, the dorsal surface of the hind paw contacts the rung, the gait cycle is incomplete, or hindlimb spasms occur during rung crossing. For the horizontal ladder test, the data included the ladder beam score (LBS, percentage of positive steps relative to total steps) and cumulative errors (total cumulative negative steps) [15]. The ladder beam score is calculated by averaging the results of five tests, while cumulative errors are computed by summing the results of five trials.

##### Rotarod Test

Testing was conducted using a rotarod apparatus (Jiangsu ScioNS Biological Technology Co., Ltd., Nanjing, China, model: SA102) The mice were placed on a rubber rotating rod (5 cm diameter). The rotation speed accelerated from 4 rpm to 40 rpm over 5 min and was maintained at the maximum speed for the subsequent 5 min. The mice underwent one adaptation test on day 1 and three formal tests on day 2, with the average of the latter used for analysis. A rest period of at least 10 min was allowed between trials. The time until falling (latency to fall) and the rotation speed at the time of falling (spin speed at fall) were recorded [16].

##### Gait Analysis

**Figure 1:**
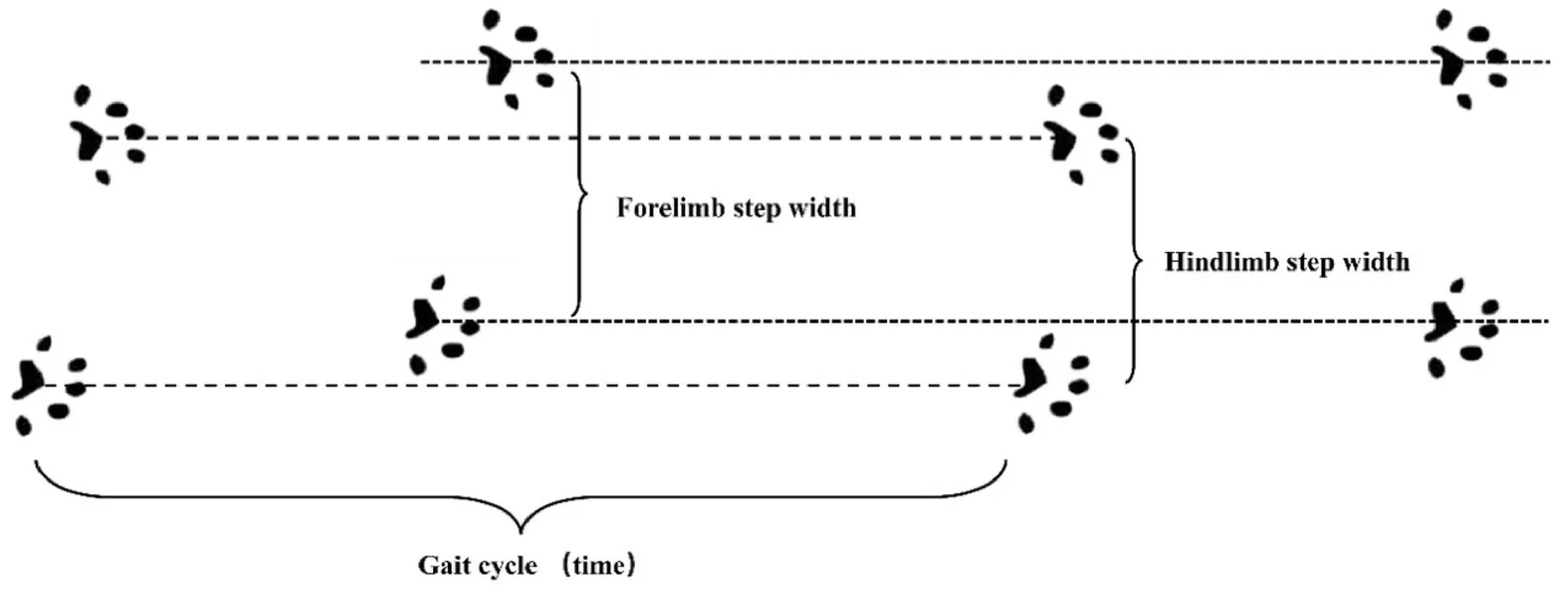
Parameters of gait analysis. Gait cycle: The time from paw contact of one limb to the next paw contact of the same limb. Forelimb step width and hindlimb step width: The horizontal distance between the centroids of the left and right forepaws (or hind paws) during simultaneous locomotion. Gait speed: The step length (distance between midpoints of consecutive paw prints of the same limb) divided by the gait cycle time. The dashed line represents the connection between the centroids of the forepaws and hindpaws.

**Figure 2:**
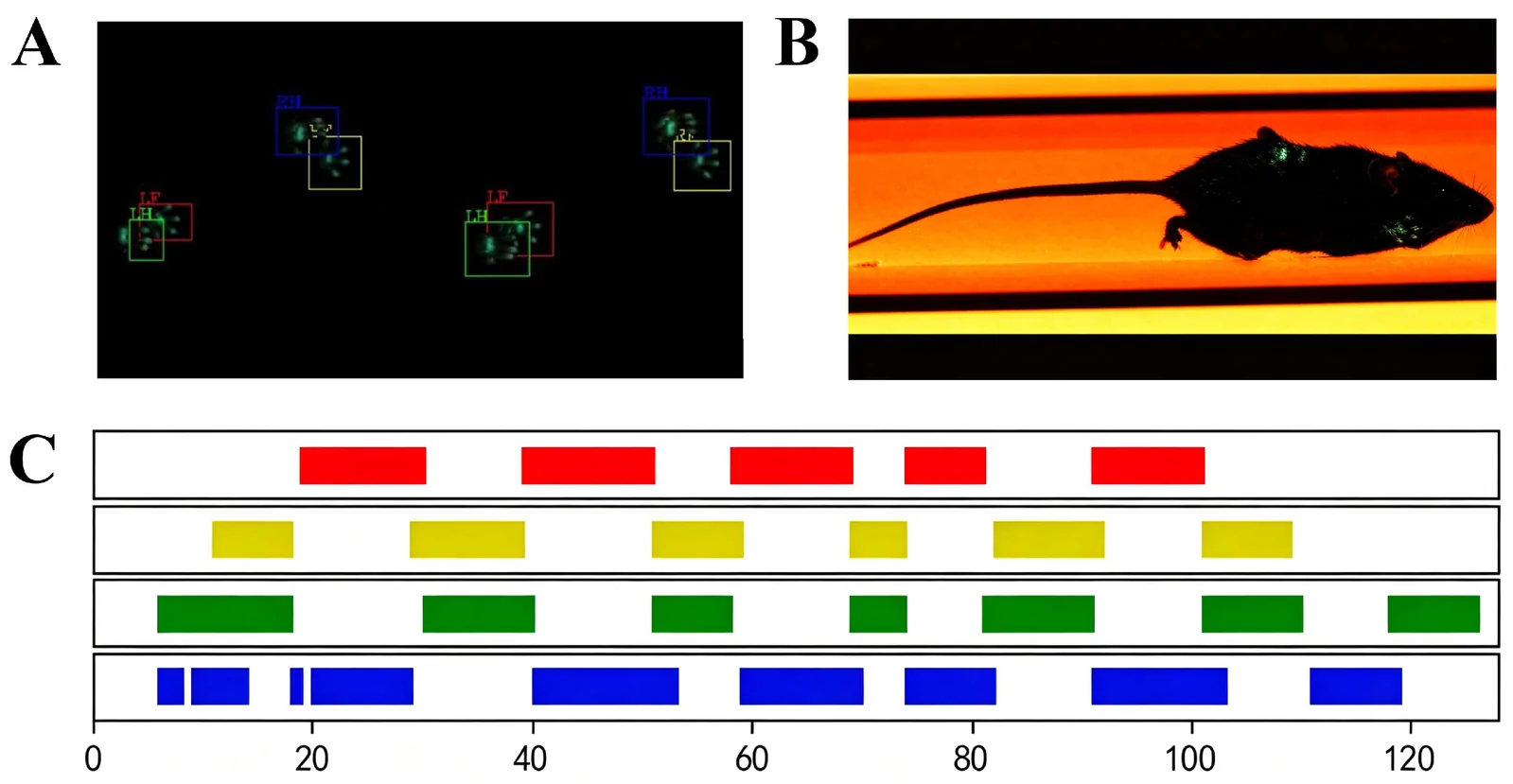
Coordination function analysis. (**A**) Paw print diagrams were generated. (**B**) Fluorescent lights at the device’s bottom illuminated the mouse’s contour, with the camera recording the movement trajectory. (**C**) The sequence of four-limb movement was recorded as the step sequence. Ipsilateral coordination was calculated as the phase of the observed paw’s stance time (e.g., right hindlimb) relative to the reference paw’s stance time (e.g., right forelimb). Contralateral coordination was the phase of the observed paw’s stance time (e.g., right hindlimb) relative to the contralateral reference paw’s stance time (e.g., left forelimb). LF: left forelimb; RF: right forelimb; LH: left hindlimb; RH: right hindlimb; Red bar: left forelimb; Yellow bar: right forelimb; Green bar: left hindlimb; Blue bar: right hindlimb.

A gait analysis system (Beijing Zhongshi Technology Co., Beijing, China, model ZS-BT/S) was used to evaluate the gait pattern. The equipment consisted of a glass walkway elevated approximately 1.5 m above the ground, two black panels perpendicular to the glass walkway, and a top plate covering the walkway, forming an enclosed animal passage. The mice traveled unidirectionally from one end of the walkway to the other, with the walkway illuminated by green LED lights. A high-speed camera positioned beneath the walkway recorded paw prints and animal contours once the mouse entered the walkway. The basic pattern of gait features was assessed by gait cycle and gait speed, which collectively reflect overall motor function. The parameter of step width was calculated as the movement stability indicator. The limb coordination parameters consisted of ipsilateral coordination and contralateral coordination, which were employed to characterize limb synchronization. The calculation for gait parameters is visually illustrated in Figure 1 and Figure 2, which detail the measurement principles for each gait metric [17,18,19].

#### 2.3.3. Histological Examination of Brain Tissue

##### c-Fos Protein Immunofluorescence Staining

After the completion of behavioral tests, 9 mice from the alkali injury group and 9 mice from the control group were randomly selected. Positive expression of c-Fos protein in the V1 and M2 regions was induced by subjecting the mice to rotarod locomotion under grating visual stimulation. The grating stimulation parameters were set as follows: horizontally moving gratings were presented on an LED liquid crystal display (LCD) screen at a moving speed of approximately 12°/s. Mice were positioned with their heads directed toward the stimulation screen at a distance of 15 cm. The spatial frequency of the grating was 0.02 cycles/°, the temporal frequency was 1 Hz, the number of accumulations was 240, the screen luminance was 20 cd/m^2^, and the contrast was 95%. The rotarod stimulation was performed at a constant speed of 10 RPM.

Following 90 min of combined stimulation, mice were deeply anesthetized and transcardially perfused. The brains were harvested and sectioned coronally at a thickness of 50 μm through the target regions V1 and M2 [20]. Immunofluorescence staining was performed on the brain sections, and the number of c-Fos-positive cells was quantified using ImageJ software (ImageJ2, UW-Madison LOCI, Madison, WI, USA).

##### Anterograde Tracing of the V1-M2 Pathway

Mice were anesthetized with sodium pentobarbital, and the scalp was disinfected and incised to expose the skull. Stereotaxic coordinates for the V1 region (AP: −3.0 mm, ML: +2.5 mm, DV: −1.0 mm) and the M2 region (AP: +1.0 mm, ML: +0.5 mm, DV: −1.0 mm) were determined according to a standard mouse brain atlas. Small burr holes were drilled through the skull over the target areas, and the dura mater was carefully removed.

To investigate the projection pathway between V1 and M2 and to perform quantitative analysis, ten healthy adult mice were used. The anterograde trans-monosynaptic tracer rAAV2/1-hSyn-Cre-WPRE-hGH polyA (300 nL) was injected into the unilateral V1 region, and rAAV2/9-hSyn-DIO-EGFP-WPREs (300 nL) were injected into the ipsilateral M2 region. After viral injection, the scalp was sutured, and a long-acting analgesic, carprofen (5 mg/kg), was administered intraperitoneally.

Three weeks after viral injection, the mice were deeply anesthetized and transcardially perfused with fixative. The brains were harvested and sectioned. To verify the accuracy of the viral injection sites, Cre protein immunostaining was performed on sections containing the V1 region. The distribution of viral expression in the V1 and M2 regions was visualized under a fluorescence microscope, and quantitative analysis was conducted in the M2 region.

##### Chemogenetic Manipulation

To chemogenetically manipulate M2 neurons that receive projections from V1, twenty visually impaired mice were used. The rAAV2/1-hSyn-Cre-WPRE-hGH polyA (300 nL) was bilaterally injected into V1, and rAAV2/9-hSyn-DIO-hM3Dq-EGFP-WPREs (300 nL) were bilaterally injected into M2. The injection procedure and stereotaxic coordinates were the same as those described for the viral tracing experiments in the preceding section. Three weeks after viral injection, the visually impaired mice were randomly divided into two groups (10 mice in the group). One group received an intraperitoneal injection of Clozapine N-oxide (CNO) solution (2 mg/kg), while the other received an equal volume of saline. Thirty minutes later, motor function was assessed in both groups using the rotarod test and the horizontal ladder test, following the same protocols as described in the motor function testing section above. After behavioral testing, positive expression of c-Fos protein in M2 neurons was induced by rotarod exercise under grating visual stimulation. The stimulation parameters and experimental procedures were identical to those used in the c-Fos immunofluorescence staining described earlier. The number of c-Fos-positive neurons among the V1-projecting M2 neurons was quantified using ImageJ software.

### 2.4. Statistical Analysis

Data analysis was performed using SPSS 24.0 software. The Shapiro–Wilk test was used to assess the normality of continuous data. Normally distributed data were compared between groups using independent-samples *t*-tests. Statistical significance was defined as *p* < 0.05. Sample size calculation was performed using PASS 11.0 software (NCSS, LLC, Kaysville, UT, USA) based on pilot study data.

## 3. Results

### 3.1. Evaluation of Corneal Morphology and Visual Function

#### 3.1.1. Changes in Cornea After Alkali Burn

**Figure 3:**
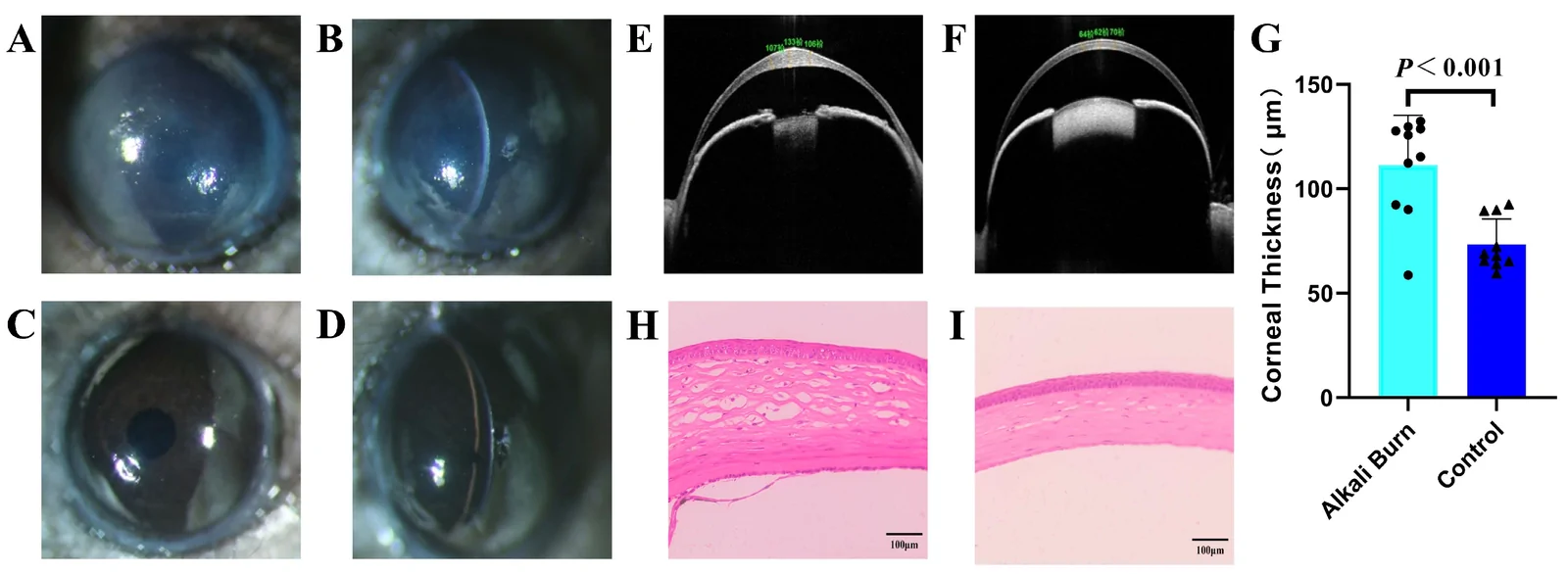
Corneal changes following alkali burn. (**A**,**B**) Slit-lamp photographs show moderate corneal opacity in the alkali burn group, while (**C**,**D**) the control group exhibits clear corneas. (**E**,**F**) Anterior segment optical coherence tomography (AS-OCT) images demonstrate significant corneal edema in the alkali burn group (**E**) compared to the control group (**F**). (**G**) Central corneal thickness (CCT) is significantly increased in the alkali burn group versus the control group (independent-samples *t*-test, *p* < 0.001). Data are presented as mean ± SD, with black dots and triangles representing individual mice. (**H**,**I**) Hematoxylin and eosin (HE) staining reveals lamellar separation of stromal collagen fibers and inflammatory cell infiltration in the alkali burn group (**H**), whereas the control group (**I**) shows normal stromal architecture.

Slit-lamp examination showed moderate corneal opacity in the alkali burn group, with a visible pupil and iris but obscured iris details (consistent with Roper-Hall grade 2). AS-OCT imaging revealed medium-to-low-intensity signals in the corneal stroma of control mice, whereas the alkali burn group exhibited hyper-reflective media signals. Central corneal thickness (CCT) was significantly greater in the alkali burn group (111.27 ± 23.94 μm) than in the control group (73.30 ± 12.30 μm); independent-samples *t*-test, t = 4.461, *p* < 0.001, d = 1.995, 95%CI = 20.086 to 55.847. HE staining demonstrated normal corneal stromal architecture with regularly arranged collagen fibers and intact endothelial cell morphology in the control group. In contrast, the alkali burn group showed lamellar separation of stromal collagen fibers, widened inter-fiber spaces, and inflammatory cell infiltration in the stroma. The results of the observation on the cornea are illustrated in Figure 3.

#### 3.1.2. Behavioral and Electrophysiological Evaluation of Visual Function

**Figure 4:**
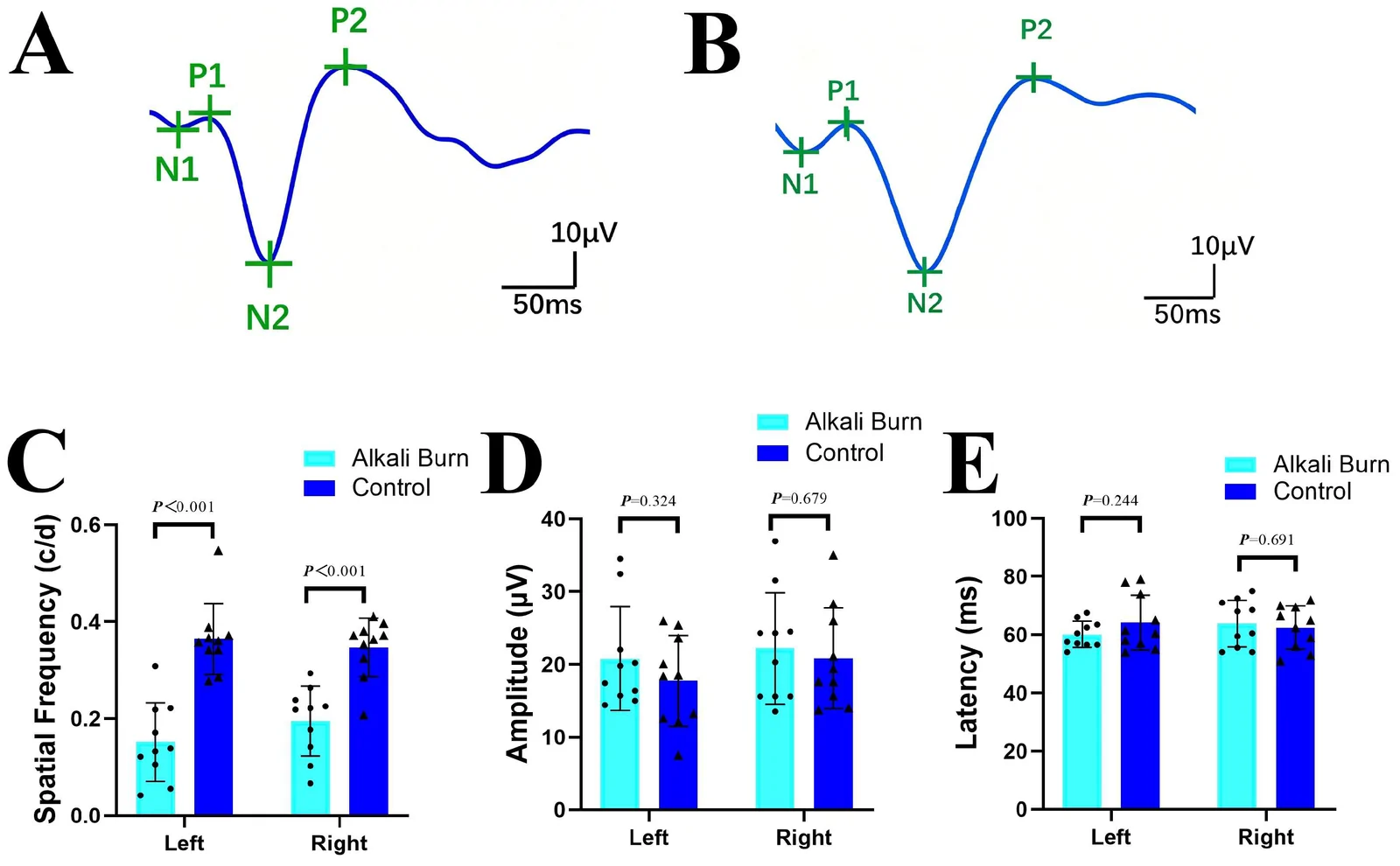
(**A**) Representative fVEP waveform from the alkali burn group. (**B**) Representative fVEP waveform from the control group. Spatial frequency (**C**) in optomotor response tests between the alkali burn and control groups. Amplitude (**D**) and latency (**E**) of P2 in flash visual evoked potentials between the alkali burn and control groups. Data are presented as mean ± SD, with black dots and triangles representing individual mice. *p*-values indicate statistically significant differences between groups (independent-samples *t*-test).

OMR tests revealed that the spatial frequency thresholds of both the right and left eyes were markedly higher in the control group (right: 0.35 ± 0.06 c/d; left: 0.36 ± 0.07 c/d) compared with the alkali burn group (right: 0.20 ± 0.07 c/d; left: 0.15 ± 0.08 c/d). Independent-samples *t*-test: right, t = 5.095, *p* < 0.001, d = 2.279, 95%CI = 0.089 to 0.214; left, t = 6.126, *p* < 0.001, d = 2.739, 95%CI = 0.139 to 0.285 (Figure 4C).

In the fVEP recordings, the amplitude and latency of the P2 wave were selected as the primary observational indicators in this study, while the recorded data of other waveform parameters (P1, N1, N2) are provided in the supplementary materials (Supplementary Table S1). No significant intergroup differences in P2 amplitudes of both the right and left eyes between the alkali burn group (right: 22.21 ± 7.65 μV; left: 20.78 ± 7.12 μV) and the control group (right: 20.84 ± 6.91 μV; left: 17.74 ± 6.25 μV). Independent-samples *t*-test: right eye, t = −0.420, *p* = 0.679, d = 0.454, 95%CI = −9.335 to 3.255; left eye, t = −1.015, *p* = 0.324, d = 0.188, 95%CI = −8.225 to 5.480 (Figure 4D). Likewise, there were no significant differences in P2 latencies of either the right or left eye between the alkali burn group (right: 63.85 ± 8.05 ms; left: 60.15 ± 4.55 ms) and the control group (right: 62.45 ± 7.41 ms; left: 64.20 ± 9.46 ms). Independent-samples *t*-test: right eye, t = −0.405, *p* = 0.691, d = 0.181, 95%CI = −8.670 to 5.870; left eye, t = 1.220, *p* = 0.244, d = 0.545, 95%CI = −3.127 to 11.227 (Figure 4E). The preserved fVEP responses confirm the structural and functional integrity of the retino-cortical pathway, whereas the reduced OMR performance demonstrates impaired visual function secondary to corneal opacification.

### 3.2. Impacts of Visual Impairment on Motor Performance

#### 3.2.1. Horizontal Ladder Test

**Figure 5:**
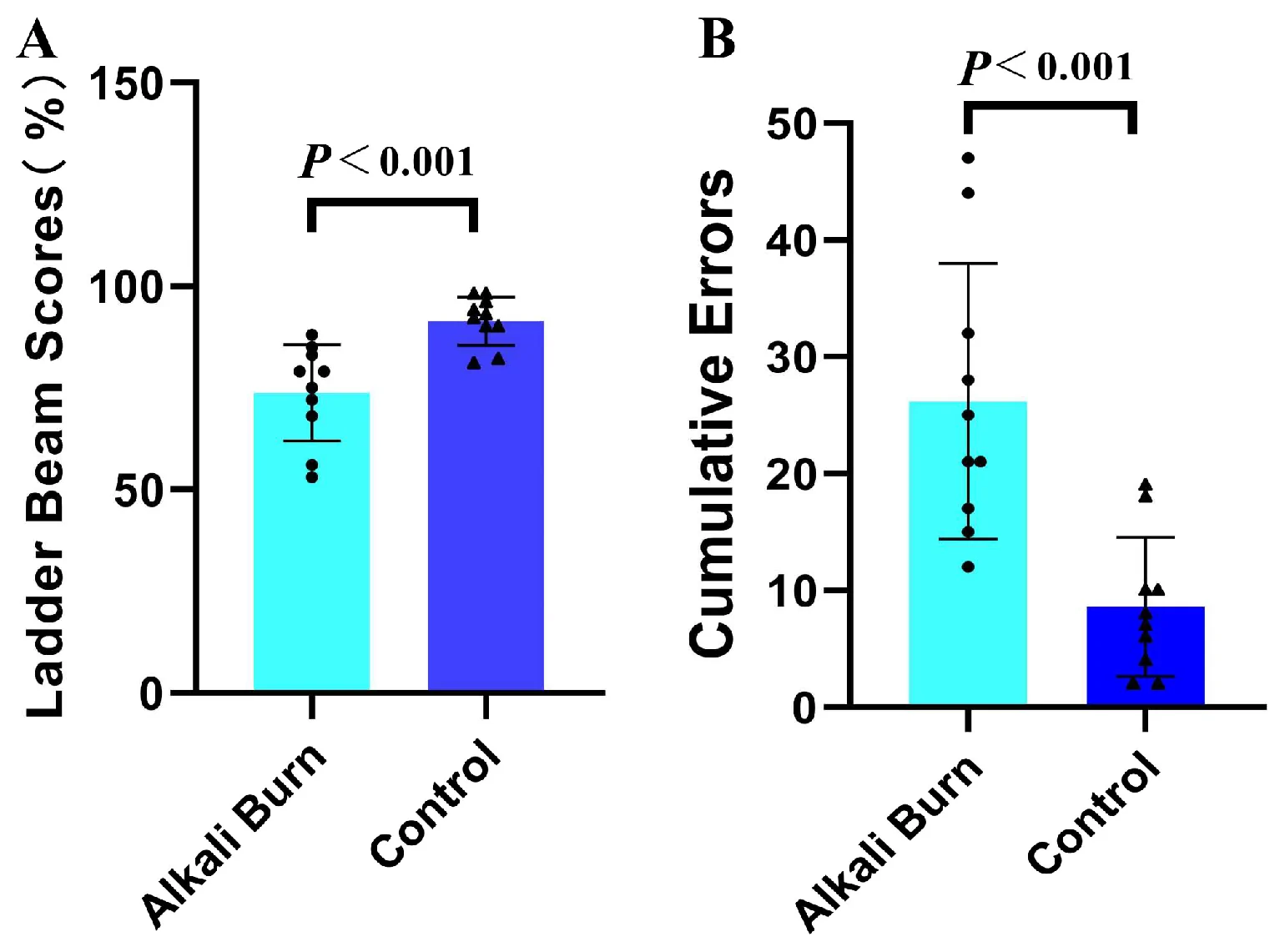
Ladder beam scores (**A**) and cumulative errors (**B**) in the horizontal ladder test between the alkali burn and control groups. Data are presented as mean ± SD, with black dots and triangles representing individual mice. *p*-values indicate statistically significant differences between groups (independent-samples *t*-test).

The LBS was significantly lower in the alkali burn group (73.80 ± 11.80%) than in the control group (91.40 ± 5.95%; independent-samples *t*-test, t = −4.211, *p* < 0.001, d = 1.883, 95%CI = 8.820 to 26.380) (Figure 5A). In contrast, the alkali burn group showed significantly higher cumulative errors (26.20 ± 11.80) compared to the control group (8.60 ± 5.95; independent-samples *t*-test, t = 4.211, *p* < 0.001, d = 1.883, 95%CI = −26.380 to −8.820) (Figure 5B). Results of the horizontal ladder test revealed that mice with visual impairment exhibited reduced motor coordination.

#### 3.2.2. Rotarod Test

**Figure 6:**
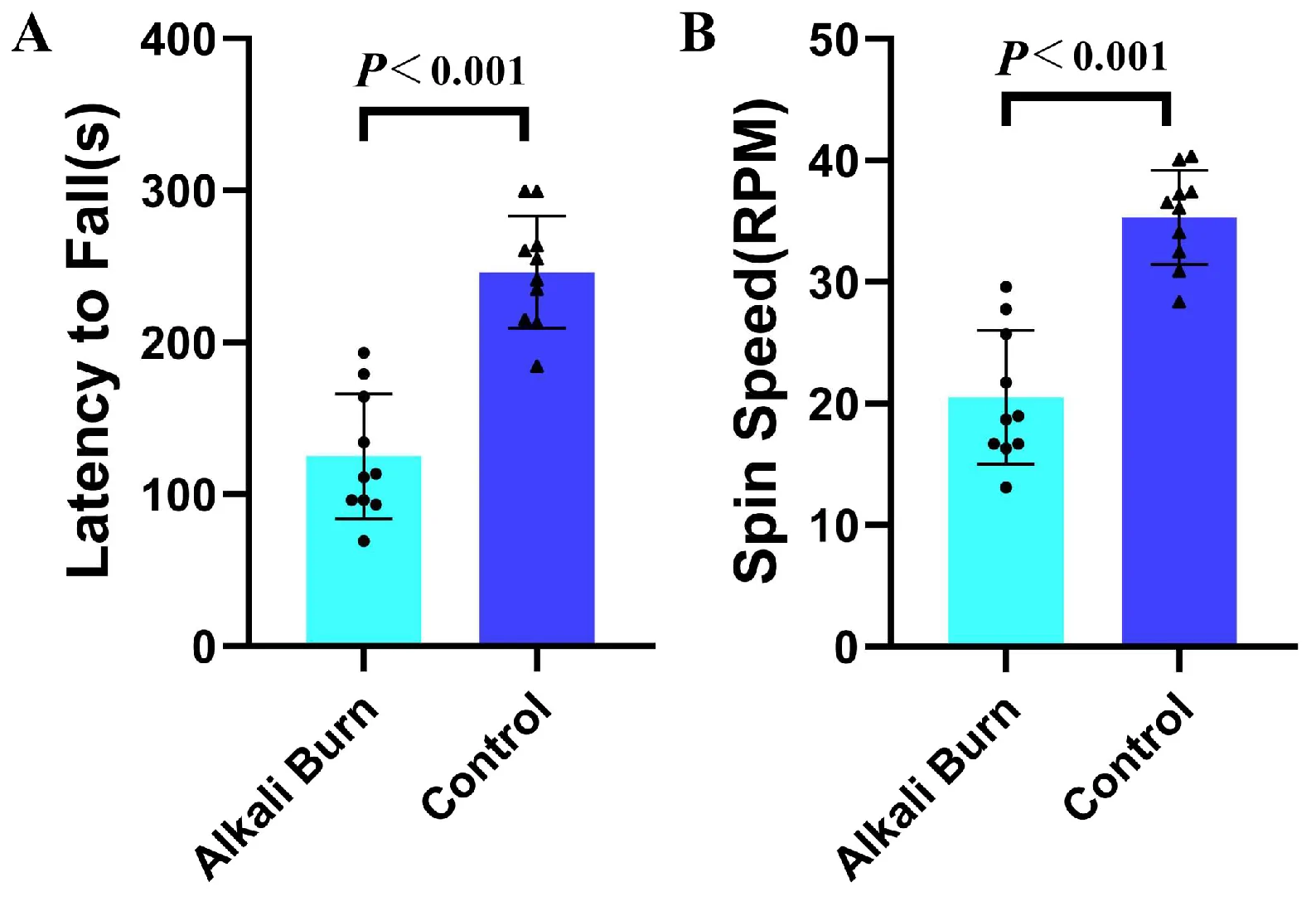
Mean values and standard deviations for (**A**) latency to fall and (**B**) spin speed at fall of the rotarod test. Data are presented as mean ± SD, with black dots and triangles representing individual mice. *p*-values indicate statistically significant differences between groups (independent-samples *t*-test).

The control mice exhibited smooth, rapid, and rhythmic stepping with stable posture and minimal paw slippage on the rotating rod. In contrast, the mice in the alkali burn group showed disrupted stepping patterns, uncoordinated limb movements, impaired postural control, and frequent paw slippage. The latency to fall was significantly shorter in the alkali burn group (125.05 ± 41.25 s) compared to the control group (246.07 ± 36.98 s; independent-samples *t*-test, t = −6.908, *p* < 0.001, d = 3.089, 95%CI = 84.217 to 157.829). (Figure 6A). The spin speed at fall was significantly lower in the alkali burn group (20.53 ± 5.49 rpm) than in the control group (35.30 ± 3.87 rpm); independent-samples *t*-test, t = −6.952, *p* < 0.001, d = 3.109, 95%CI = 10.309 to 19.238 (Figure 6B).

#### 3.2.3. Gait Analysis

**Figure 7:**
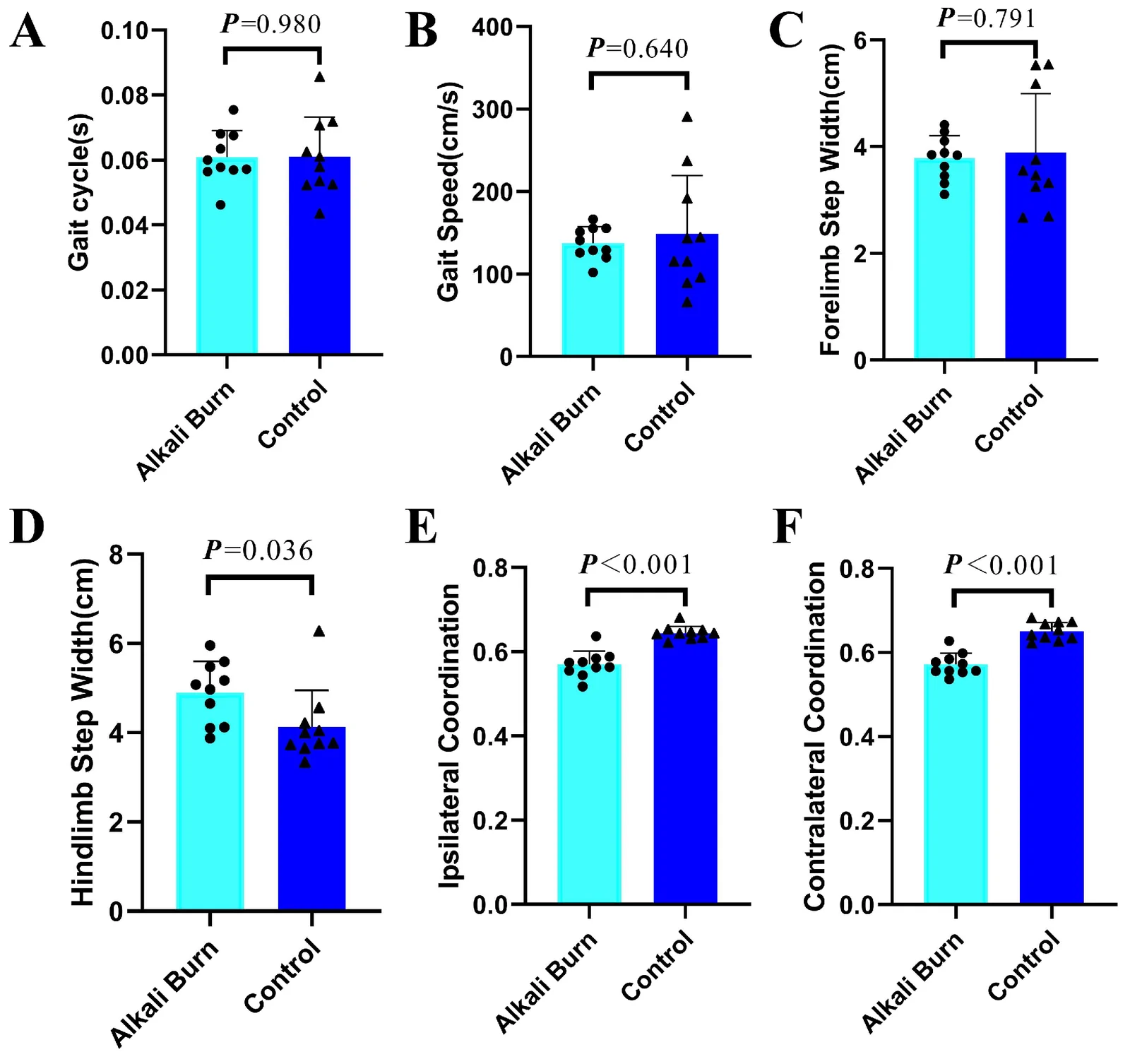
Gait analysis results between the alkali burn and control groups. (**A**) Gait cycle, (**B**) gait speed, and (**C**) forelimb step width showed no significant differences between groups. (**D**) Hindlimb step width was significantly higher in the alkali burn group than in the control group. (**E**) Ipsilateral coordination score and (**F**) contralateral coordination scores were significantly lower in the alkali burn group than in the control group. Data are presented as mean ± SD, with black dots and triangles representing individual mice. Statistical significance was determined by independent-samples *t*-test, and *p*-values are indicated above each panel.

No significant differences were observed in gait cycle (alkali burn group: 0.06 ± 0.01 s vs. control: 0.06 ± 0.01 s, *p* = 0.980, d = 0.011, 95%CI = −0.010 to 0.010) or gait speed (alkali burn group: 137.55 ± 19.78 cm/s vs. control: 148.77 ± 70.84 cm/s, independent-samples *t*-test, t = −0.482, *p* = 0.640, d = 0.216, 95%CI = −40.342 to 62.777), indicating preserved overall motor rhythm in visually impaired mice. The alkali burn group showed significantly increased hindlimb step width (4.90 ± 0.70 cm vs. 4.13 ± 0.82 cm, independent-samples *t*-test, t = −2.262, *p* = 0.036, d = 1.012, 95%CI = −1.489 to −0.055), suggesting compensatory adjustment. No significant difference was observed in forelimb step width between the two groups (alkali burn group: 3.79 ± 0.42 cm vs. control group: 3.89 ± 1.11 cm; independent-samples *t*-test, t = −0.272, *p* = 0.791, d = 0.122, 95%CI = −0.718 to 0.921). Ipsilateral coordination score (0.57 ± 0.03 vs. 0.64 ± 0.02, independent-samples *t*-test, t = −6.666, *p* < 0.001, d = 2.981, 95%CI = 0.051 to 0.097) and contralateral coordination score (0.57 ± 0.03 vs. 0.65 ± 0.02, independent-samples *t*-test, t = −7.267, *p* < 0.001, d = 3.250, 95%CI = 0.056 to 0.101) were significantly lower in the alkali burn group, reflecting disrupted limb synchronization. Results of gait analysis are shown in Figure 7.

### 3.3. Role of the V1-M2 Circuit in Visual Impairment-Induced Balance and Coordination Dysfunction

#### 3.3.1. Altered c-Fos Expression Was Observed in the V1 and M2 Brain Regions of Mice with Motor Dysfunction Induced by Visual Impairment

**Figure 8:**
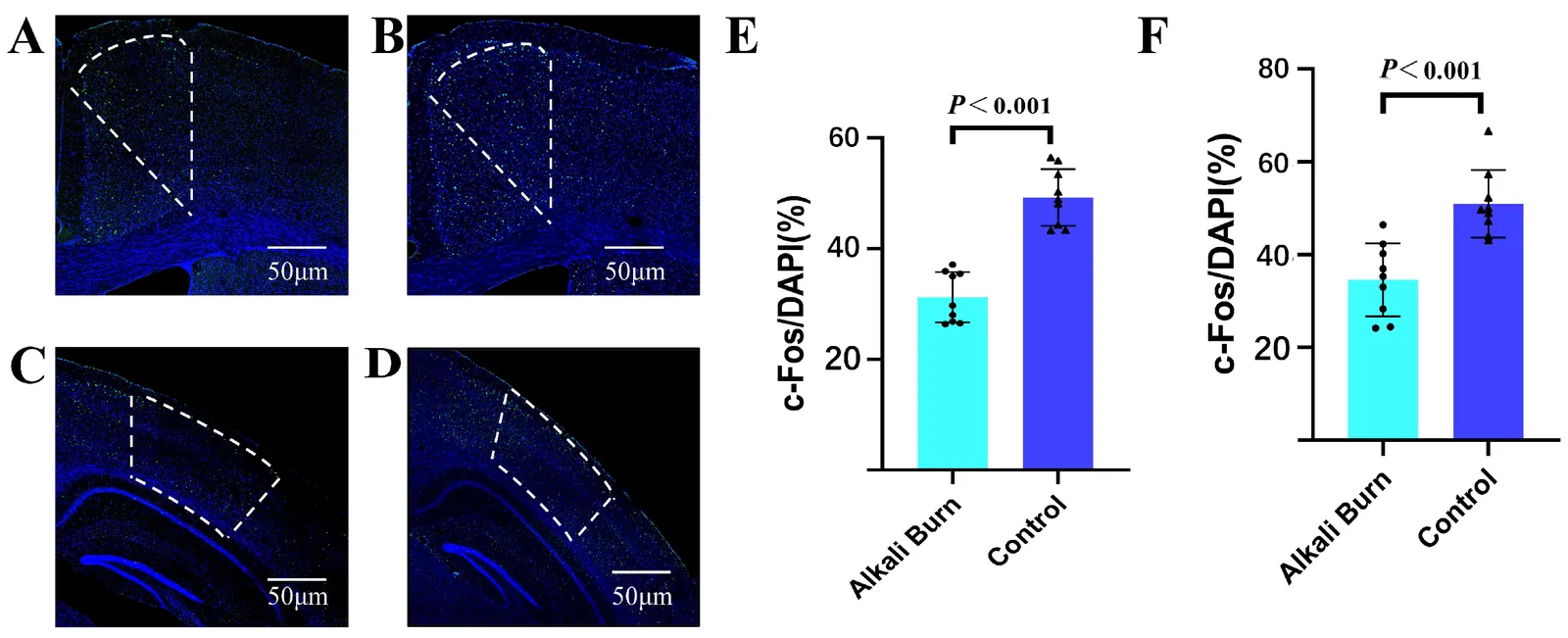
Representative immunofluorescence images of c-Fos staining and quantitative analysis of c-Fos-positive cells in brain regions V1 and M2. (**A**) Representative c-Fos staining images of the M2 region in the alkali burn group, White dashed lines outline the M2 region. AP: 1.0. (**B**) Representative c-Fos staining images of the M2 region in the control group, White dashed lines outline the M2 region. AP: 1.0. (**C**) Representative c-Fos staining images of the V1 region in the alkali burn group, White dashed lines outline the V1 region. AP: −3.0. (**D**) Representative c-Fos staining images of the V1 region in the control group, White dashed lines outline the V1 region. AP: −3.0. Green fluorescence indicates c-Fos+ cells, while blue fluorescence corresponds to DAPI-counterstained nuclei. (**E**) c-Fos^+^ cells in V1 were quantified relative to DAPI, and (**F**) c-Fos^+^ cells in M2 were quantified relative to DAPI. Levels were significantly lower in the alkali burn group than in the control group. Data are presented as mean ± SD, with black dots and triangles representing individual mice. Statistical significance was determined by independent-samples *t*-test, and *p*-values are indicated above each panel.

Comparison of c-Fos expression in V1 and M2 between the two groups revealed that both regions were activated by combined visual and motor stimulation, as shown in Figure 8A–D. The percentage of c-Fos positive cells in V1 of the alkali burn group was significantly decreased (31.23% ± 4.57% vs. 49.26% ± 5.14%, independent-samples *t*-test, t = 7.862, *p* < 0.001, d = 3.706, 95%CI = 0.132 to 0.229). The percentage of c-Fos-positive cells in M2 of the alkali burn group was significantly decreased (34.57% ± 7.84% vs. 50.96% ± 7.24%, independent-samples *t*-test, t = 4.608, *p* < 0.001, d = 2.172, 95%CI = 0.089 to 0.239). The quantitative results are presented in Figure 8E,F. The results showed that both V1 and M2 are involved in mediating motor dysfunction induced by visual impairment, and the activation levels of the two brain regions exhibited synchronous changes.

#### 3.3.2. Existence of a V1-M2 Neural Circuit Between the V1 and M2 Brain Regions

**Figure 9:**
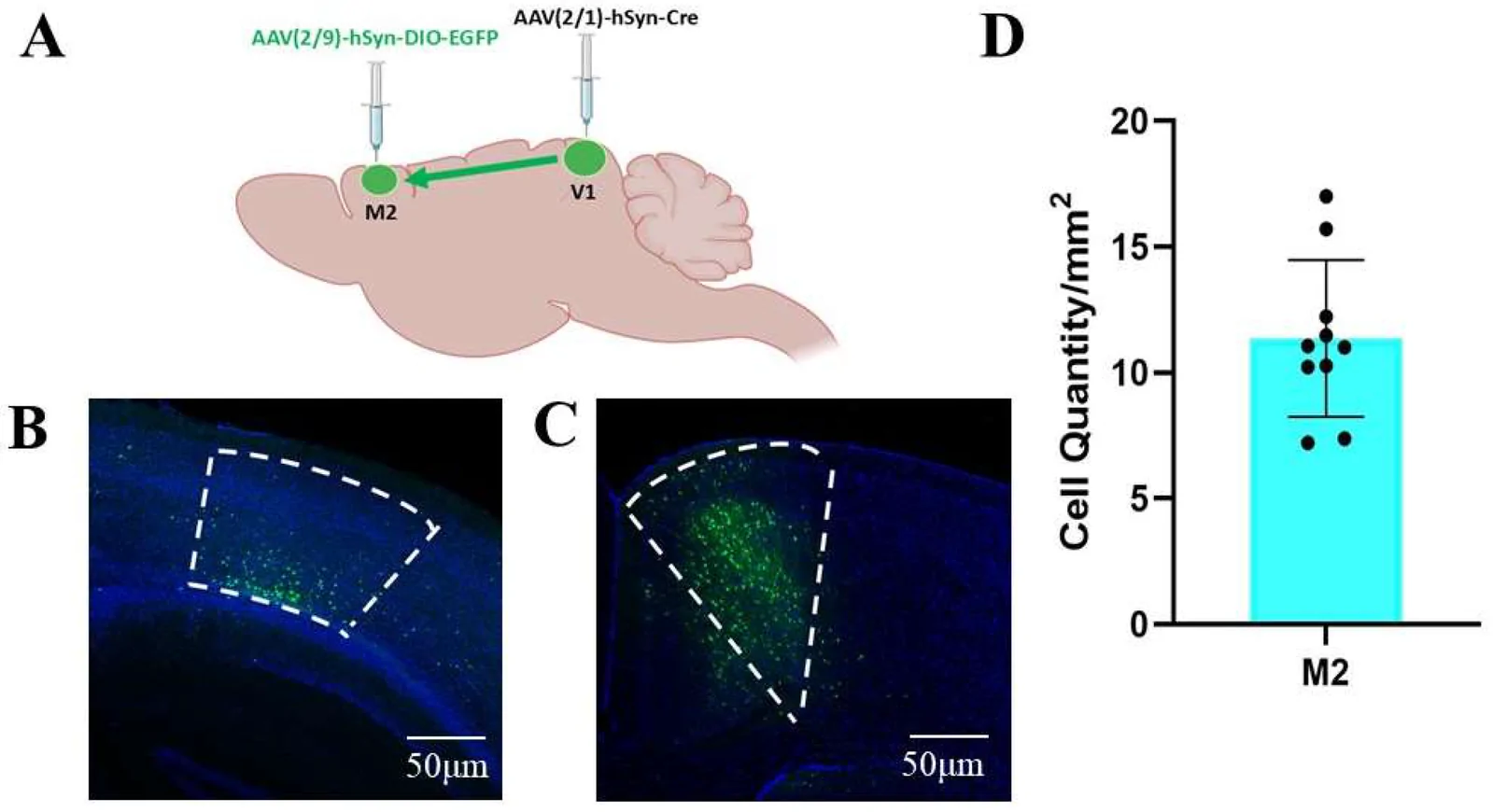
Anterograde tracing of the V1-M2 circuit and quantitative analysis of positive neurons in the M2 region. (**A**) Schematic diagram of anterograde tracer virus injection targeting the V1-M2 pathway. Arrows indicate the direction of viral spread from V1 to M2. (**B**) Representative image of the injection site in the V1 region showing Cre immunopositive neurons. EGFP^+^ neurons were labeled by CRE-mediated recombination (green), and nuclei were counterstained with DAPI (blue). White dashed lines outline the V1 region. Scale bar: 50 μm. (**C**) Representative micrograph of EGFP^+^ neurons in the M2 region. EGFP^+^ neurons were visualized in green, and nuclei were visualized in blue. White dashed lines outline the M2 region. Scale bar: 50 μm. (**D**) Quantitative analysis of the density of EGFP^+^ neurons in the M2 region. Data are expressed as mean ± SD, and each black dot represents an individual mouse.

To investigate the projection pathway between V1 and M2 and to perform quantitative analysis, we employed an anterograde trans-monosynaptic viral tracing strategy: rAAV2/1-hSyn-Cre was injected into the unilateral V1 region, and rAAV2/9-hSyn-DIO-EGFP was injected into the ipsilateral M2 region (Figure 9A). Three weeks after viral injection, the accuracy of the injection site was verified by immunofluorescence staining of Cre protein in the V1 region. The results showed that Cre-positive neurons were clearly visible in the V1 region, confirming successful transduction of the target brain area (Figure 9B). In the M2 region, abundant EGFP^+^ neurons were observed, indicating successful labeling of M2 neurons that receive projections from V1 (Figure 9C). Quantitative analysis revealed that the density of EGFP^+^ neurons in the M2 region was 11.36 ± 3.12 cells/mm^2^ (Figure 9D), further confirming the existence of a stable monosynaptic projection between V1 and M2.

#### 3.3.3. Activation of the V1-M2 Circuit Ameliorates Motor Dysfunction Induced by Visual Impairment

**Figure 10:**
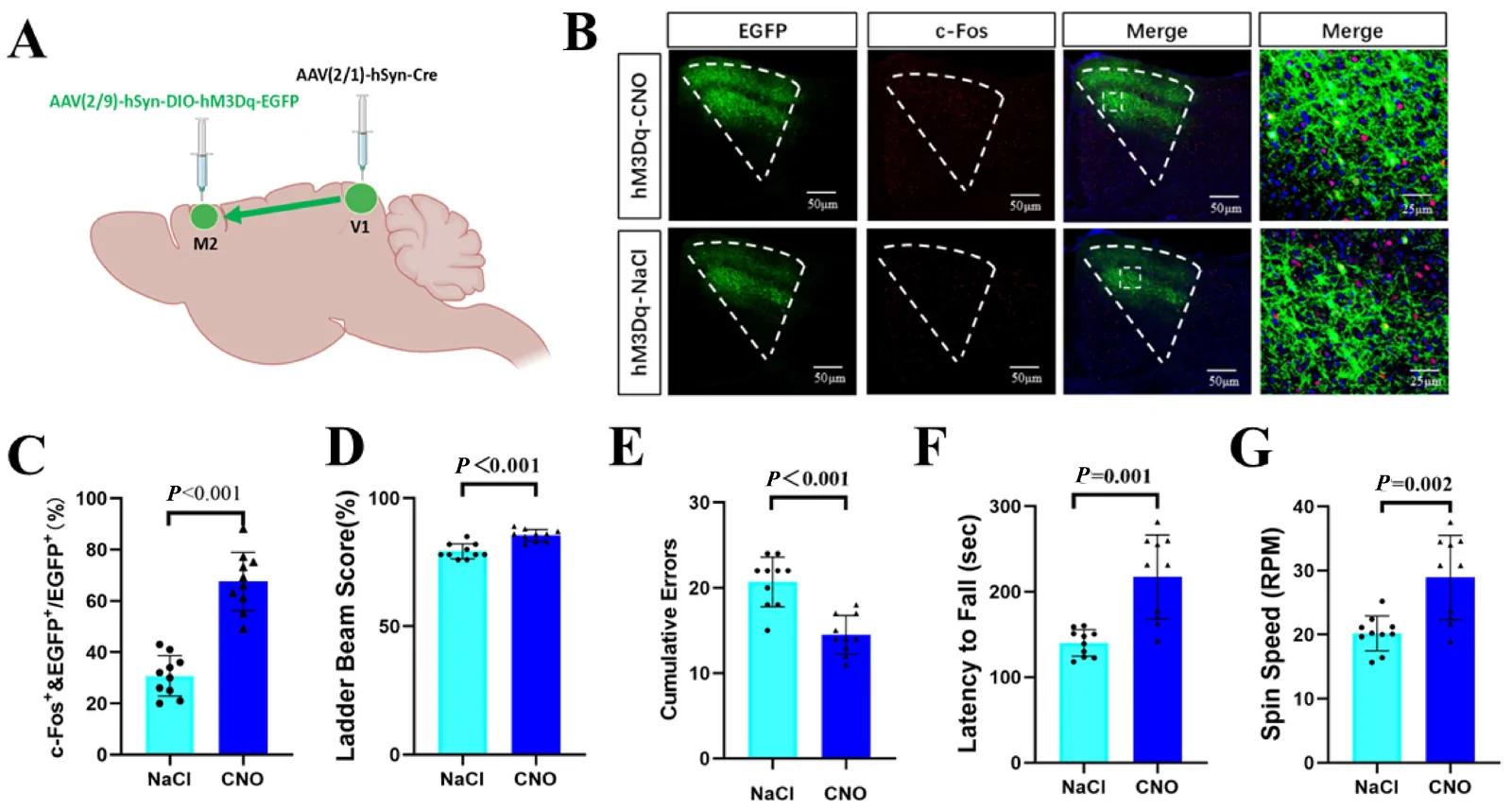
Chemogenetic activation of the V1-M2 circuit ameliorates motor dysfunction in visually impaired mice. (**A**) Schematic Diagram of Viral Injection for Chemogenetic Manipulation of the V1-M2 Circuit. Arrows indicate the direction of viral spread from V1 to M2. (**B**) Representative fluorescence images showing hM3Dq-EGFP^+^ neurons (green) and c-Fos^+^ neuron expression (red) in the M2 region following intraperitoneal injection of CNO (upper) or saline (lower) in visually impaired mice. Merge indicates multi-channel fluorescence overlay. White dashed lines outline the M2 region; white boxes indicate magnified areas. Scale bars: 50 μm (overview), 25 μm (inset). (**C**) Quantification of the percentage of hM3Dq-EGFP^+^ neurons coexpressing c-Fos^+^ neurons in the M2 region in the CNO group versus the NaCl group. (**D**) Ladder beam scores and (**E**) Cumulative errors in the horizontal ladder test; (**F**) Latency to fall and (**G**) Spin speed at fall in the rotarod test. Data are presented as mean ± SD, with black dots and triangles representing individual mice. *p*-values indicate statistically significant differences between groups as determined by independent-samples *t*-test.

In visually impaired mice, AAV2/1-hSyn-Cre virus was injected into V1, and AAV2/9-hSyn-DIO-hM3Dq-EGFP virus was injected into M2 (Figure 10A). This was performed to specifically label M2 neurons receiving projections from V1 and render them activatable by CNO. Three weeks after virus injection, the mice were randomly divided into two groups and received intraperitoneal injections of either CNO solution or an equal volume of saline, respectively. Representative images of c-Fos activation in EGFP-labeled neurons in the M2 region (Figure 10B). Quantitative analysis of c-Fos activation levels in EGFP-labeled neurons in the M2 region. The c-Fos^+^&EGFP^+^/EGFP^+^ was significantly lower in the NaCl group (30.64 ± 7.79%) than in the CNO group (67.59 ± 11.58%; independent-samples *t*-test, t = −8.376, *p* < 0.001, d = 3.746, 95%CI = −0.462 to −0.277) (Figure 10C). Motor function was then assessed in both groups. The LBS was significantly lower in the NaCl group (79.30 ± 2.91%) than in the CNO group (85.50 ± 2.27%; independent-samples *t*-test, t = −5.312, *p* < 0.001, d = 2.376, 95%CI = −8.652 to −3.748) (Figure 10D). In contrast, the NaCl group showed significantly higher cumulative errors (20.70 ± 2.91) compared to the CNO group (14.50 ± 2.27; independent-samples *t*-test, t = 5.312, *p* < 0.001, d = 2.376, 95%CI = 3.748 to 8.652) (Figure 10E). The latency to fall was significantly shorter in the NaCl group (140.14 ± 15.60 s) compared to the CNO group (217.37 ± 49.24 s; independent-samples *t*-test, *t* = 4.728, *p* < 0.001, d = 2.114, 95%CI = 41.185 to 113.261) (Figure 10F). The spin speed at fall was significantly lower in the NaCl group (20.20 ± 2.72 rpm) than in the CNO group (28.93 ± 6.58 rpm); independent-samples *t*-test, *t* = 3.879, *p* < 0.002, d = 1.735, 95%CI = 3.828 to 13.639 (Figure 10G).

## 4. Discussion

Alkali corneal burn in mice is a model capable of inducing visual impairment of varying severities, and we therefore employed this model in the present study to investigate visuomotor integration. In the present study, we specifically induced moderate visual impairment in mice, corresponding to Roper-Hall grade 2 corneal alkali burn. This grade of injury induces corneal scarring without perforation, leading to a consistent moderate reduction in visual function [21]. Functional assessments were conducted on day 14 post-alkali burn, a time point at which corneal edema had mostly resolved [22]. We performed spontaneous blink counting as an indirect indicator of ocular discomfort, and found no significant inter-group difference in blink frequency (methods and results are shown in Supplementary Table S2). In line with published evidence, this result suggests that acute inflammation and ocular pain are markedly alleviated at this stage [23].

In this study, we combined the optomotor response assay and flash visual evoked potential recording to evaluate visual function in mice. The optomotor test showed that spatial frequency thresholds corresponding to both eyes were significantly lower in the corneal alkali burn group than in the control group, indicating impaired visual resolution and the presence of visual dysfunction. For fVEP, the P2 wave serves as a core parameter for assessing the functional integrity of the visual pathway from the retina to the primary visual cortex [24,25]. No statistically significant intergroup differences were observed in either P2 latency or amplitude for either stimulation side. The results from our visual function assessments indicate that this model specifically induces degradation of visual image quality caused by opacity of the anterior segment refractive media, while preserving the overall structural and functional integrity of the retino-cortical pathway. This distinction is fundamental to interpreting the subsequent findings. The observed motor phenotypes and circuit alterations represent adaptive responses of the mature brain to degraded spatial visual input, rather than direct consequences of organic visual pathway injury.

Visual dysfunction impairs motor balance and coordination, yet the magnitude of this effect requires quantitative evaluation. In the present study, we employed the horizontal ladder test and rotarod test to assess motor balance and coordination in mice. The results demonstrated that mice with corneal scarring induced by alkali burn exhibited significant deficits in these motor functions. Previous studies have found that mice with hereditary visual impairment showed significantly lower balance and coordination performance in the rotarod test than control mice [7]. Notably, Voller et al. reported no significant difference in balance function assessed via the rotarod test between 3-month-old C3H mice with retinal degeneration and wild-type mice; however, after whisker removal to eliminate tactile compensation, balance function was significantly impaired in C3H mice with retinal degeneration [26]. The central nervous system (CNS) maintains motor balance and coordination through the integration of multisensory information. When visual input is reduced, other sensory systems typically mount compensatory responses to mitigate functional deficits. In our corneal alkali burn model, visual impairment occurs within a relatively short time frame, during which compensatory mechanisms are not yet fully established. Consequently, overall motor balance and coordination capacity are significantly reduced. So, our observations provide supplementary evidence that visual impairment severely compromises motor balance and coordination, and suggest the potential utility of the mouse model of corneal alkali burn-induced visual impairment for investigating sensorimotor integration.

Gait analysis revealed no statistically significant differences in gait cycle or gait speed between visually impaired mice and control mice. This finding is consistent with the features of our mouse model. The moderate corneal opacification (Roper-Hall grade 2) preserves partial residual visual input, and fVEP recordings confirm intact basic function of the retino-cortical visual pathway. Sustained visual afferent signals are thus sufficient to maintain the fundamental spatio-temporal characteristics of gait. Similarly, there was no statistically significant difference in forelimb stride width between the two groups. Rodent quadrupeds rely primarily on their forelimbs for deceleration and braking [27,28]. Consistent with this, no statistically significant intergroup differences were observed in walking speed and gait cycle. These findings confirm that visual impairment does not disrupt braking control during locomotion, resulting in comparable overall movement rhythm and traveling speed between the two groups. In contrast, mice with visual impairment showed significant impairments in both ipsilateral and contralateral coordination. The disruption of both parameters suggests that the precise coupling of limb movements depends more strongly on real-time visual feedback than on basic gait rhythm, and is therefore more susceptible to reduced visual input quality. Meanwhile, the increased hindlimb step width can be interpreted as a compensatory adaptation to impaired coordination [29,30]. The previous study using mouse models of retinitis pigmentosa has revealed that gene therapy preserving only a subset of photoreceptors is sufficient to maintain normal motor performance in the Morris water maze test, suggesting that basic motor function is not sensitive to non-severe visual impairment [8]. In line with this finding, our results demonstrate that balance and coordination function is more susceptible to the quality of visual input, representing a phenotype of selective motor impairment resulting from diminished real-time navigation of vision. This phenotypic profile further supports that the mild-to-moderate, acquired visual image quality degradation induced by corneal alkali burn specifically impairs high-precision vision-guided motor tasks, while leaving basic locomotor function largely intact.

Visual impairment interferes with cerebral sensorimotor integration, which in turn compromises motor function. To explore cerebral functional alterations correlated with motor deficits in response to visual impairment, histological analyses are employed to assess neuronal activation and select the cerebral regions related to the changes in sensorimotor integration underlying the impacts. The expression level of c-Fos protein can effectively characterize the neuronal activation intensity of relevant brain regions under specific behavioral paradigms. V1 encodes basic visual features including orientation, spatial frequency, and motion direction, and integrates afferent signals from subcortical structures to regulate higher-order visual functions [31], making it a core target brain region for c-Fos expression assessment in vision-related research. As a key node in the neural circuit for the flexible regulation of voluntary behavior, the activation status of M2 is of great research value for deciphering the mechanism of sensorimotor integration. Previous studies have confirmed that M2 receives somatosensory and motor-related inputs, and inactivation of this brain region significantly disrupts the transformation of sensorimotor signals [32,33].

Our results showed that the proportion of c-Fos^+^ cells in both the V1 and M2 regions was significantly reduced in visually impaired mice. As the primary cortical region for visual information processing, the decreased c-Fos expression in V1 aligns with weakened visual afferent input caused by corneal opacification. The parallel reduction in neuronal activation in M2, a high-order premotor region involved in sensorimotor integration, indicates that reduced visual input is accompanied by downregulated activity in motor-related cortical regions. The synchronized activity changes in these two regions suggest a potential functional linkage between V1 and M2 that may be involved in visual impairment-related motor dysfunction.

To verify the anatomical basis of this potential linkage, we employed anterograde trans-monosynaptic viral tracing to verify the existence of stable neural circuit projections between the mouse V1 and M2 brain regions. Viral tracing demonstrated monosynaptic projections from V1 neurons to M2, where these inputs form stable synaptic connections. Consistent with the prior work of Hovde [6]. Together, these data support the existence of a structural substrate for the V1-M2 visuomotor circuit.

To further explore the role of the V1-M2 circuit in visual impairment-induced motor deficits, we used chemogenetic tools to specifically activate the V1-M2 circuit. Following CNO administration, c-Fos expression was significantly increased in the M2 region. Combined with anatomical tracing evidence, these results indicate that targeted activation of the V1-M2 pathway has the potential to ameliorate balance and coordination deficits in model mice. However, the mechanism by which enhancing circuit activity compensates for visual information degradation remains to be fully elucidated. Given that corneal opacity primarily reduces the signal-to-noise ratio of visual input rather than abolishing visual pathway function, we propose two non-mutually exclusive mechanisms underlying the therapeutic effect of V1-M2 pathway activation. First, enhanced activity of M2 neurons may directly boost the output strength of the motor pathway. Second, the chemogenetic activation may lower the activation threshold of M2 neurons, compensating for insufficient drive caused by degraded visual signals. In this way, the circuit becomes more efficient at utilizing available visual signals to guide motor behavior. However, we acknowledge that alternative interpretations cannot be excluded by the current data. Although the present study demonstrates that activation of the V1-M2 pathway is sufficient to improve motor behavioral performance, chemogenetic activation may increase motor circuit excitability, enhance behavioral arousal, or improve motor readiness. Any of these factors could independently lead to improved motor performance; thus, these alternative mechanisms remain plausible. Future studies employing chemogenetic manipulations combined with electrophysiological recordings will be required to disentangle these potential mechanisms and clarify the precise mechanisms by which modulation of the V1-M2 pathway exerts its effects.

The present study has several limitations that should be acknowledged. First and foremost, the corneal alkali burn model employed in this study is inherently an ocular surface trauma model characterized by refractive media opacity, rather than a generic model of visual impairment. The visual dysfunction induced by this model specifically presents as adult-onset, mild-to-moderate degradation of visual image quality originating from peripheral optical media, with preserved structural and functional integrity of the retina and visual pathway. Accordingly, the findings of this study are only applicable to this specific category of visual dysfunction and cannot be directly generalized to other forms of visual impairment such as inherited retinal degeneration, optic nerve injury, glaucoma, or developmental amblyopia. Furthermore, the present study only established a single severity grade of corneal alkali burn injury and lacks a systematic comparison across visual impairment models with different injury severities and anatomical lesion sites. The differential impacts of distinct types of visual impairment on motor function remain to be further characterized. In addition, this model is accompanied by a local inflammatory response and tissue healing process following corneal trauma. Although we strictly controlled the injury severity to minimize systemic and behavioral interference, the potential impact of mild ocular discomfort on motor performance cannot be completely excluded. Second, this study only conducted observations on day 14 after corneal injury, which only captures motor function alterations in the subacute phase of visual impairment. The long-term trajectory of motor function changes in the chronic phase, as well as the temporal pattern of multisensory compensatory adaptation and the corresponding neural circuit remodeling mechanisms, remains to be fully elucidated. Future studies with extended observation periods will not only help to better control for potential confounding effects of chronic inflammation and pain on motor function outcomes, but also clarify the dynamic progression of motor dysfunction and compensatory adaptation following visual input degradation. Third, only male mice were used for the measurements in the present study, with female mice excluded from data analysis. Therefore, potential confounding effects of sex differences on experimental outcomes cannot be ruled out in this work. Future studies should incorporate observations on female mice to explore sex-related discrepancies and improve the generalizability of the experimental conclusions. Fourth, c-Fos serves as an indirect marker of neuronal activity. Electrophysiological recordings should be incorporated in subsequent experiments to assess circuit function, which will further elucidate the role of the V1-M2 circuit in vision-guided motor behaviors. Fifth, the present study only focused on the analysis of target brain regions and did not examine other visuomotor-associated areas, including the posterior parietal cortex, superior colliculus, and thalamus. These regions are all more involved in high-order sensorimotor integration, and their functional mechanisms under the experimental paradigm of this study remain unclear. Future research will further investigate these brain regions to deepen our understanding of the neural circuits underlying visuomotor regulation.

## 5. Conclusions

Our study demonstrates that moderate visual impairment induced by corneal alkali burn impairs balance and coordination in mice, an effect associated with reduced neuronal activation of the V1-M2 circuit. Optomotor and flash VEP tests confirm that the visual dysfunction arises from anterior segment refractive media opacity, with the retino-cortical conduction pathway remaining functionally intact. Chemogenetic activation of the V1-M2 circuit ameliorates motor deficits in visually impaired mice, providing preliminary evidence supporting its potential mediating role. Future studies employing targeted circuit manipulation approaches are warranted to further validate the causal relationship and to deepen mechanistic understanding of visuomotor integration.

## Data Availability

The raw data supporting the conclusions of this article will be made available by the authors on request.

## Supplementary Materials

The following supporting information can be downloaded at: https://www.mdpi.com/article/10.3390/brainsci16080774/s1, Table S1: fVEP Test Result; Table S2: Eye Blink Count Test Result.

## Abbreviations

AS-OCT	Anterior Segment Optical Coherence Tomography
LBS	Ladder Beam Score
LGN	Lateral Geniculate Nucleus
V1	Primary Visual Cortex
M1	Primary Motor Cortex
M2	Secondary Motor Cortex
CCT	Central corneal thickness
OCTA	Optical Coherence Tomography Angiography
OMR	Optomotor Response
fVEP	flash Visual Evoked Potential
CNS	Central Nervous System
EEG	Electroencephalography
