# Supplementary material for: Association of Altered V1-M2 Neuronal Activation with Balance and Coordination Deficits in a Corneal Alkali Burn Mouse Model of Visual Impairment
Source: Brain Sci. 2026 Jul 23;16(8):774. doi: 10.3390/brainsci16080774 (PMC13511566; doi:10.3390/brainsci16080774)
Supplement: Supplementary file 1 [file brainsci-16-00774-s001.zip › brainsci-4250193-supplementary.pdf]

Table S1. fVEP Test Result.

	Left N1 latency （ms）	Right N1 latency （ms）	Left N1 amplitude （uv）	Right N1 amplitude （uv）	Left P1 latency （ms）	Right P1 latency （ms）	Left P1 amplitude （uv）	Right P1 amplitude （uv）	Left N2 latency （ms）	Right N2 latency （ms）	Left N2 amplitude （uv）	Right N2 amplitude （uv）
Control	8.85±2.04	6.8±3.51	-1.47±0.61	-1.58±1.37	21.25±2.25	18.4±4.59	3.93±1.7	3.64±3.03	38.6±2.45	35.8±3.57	-11.81±4.2	-10.77±4.9
Alkali	10.25±2.9	7.85±5	-1.63±1.05	-1.89±0.94	22.3±2.76	20.5±4.89	4.18±3.12	4.51±2.26	39±4.37	38.1±4.43	-11.55±6.79	-11.51±5.79
t	-1.249	-0.543	0.418	0.589	-0.932	-0.990	-0.223	-0.728	-0.253	-1.278	-0.103	0.309
P	0.228	0.594	0.681	0.563	0.364	0.335	0.826	0.476	0.803	0.217	0.919	0.761
d	0.558	0.243	0.187	0.263	0.417	0.443	0.100	0.326	0.113	0.572	0.046	0.138
95%CI	-3.756,0.956	-5.11,3.01	-0.644,0.964	-0.797,1.417	-3.417,1.317	-6.556,2.356	-2.61,2.11	-3.38,1.64	-3.725,2.925	-6.081,1.481	-5.567,5.047	-4.299,5.779

Eye Blink Count Test

Spontaneous blink counts of mice in both groups were measured on postoperative day 14. Each mouse was placed in an observation chamber measuring 24 × 45 × 20 cm (length × width × height) and allowed to acclimate to the environment for 15 minutes. Thereafter, an investigator used a manual counter to record the total number of spontaneous bilateral blinks in real time over a 5-minute observation period.

Table S2. Eye Blink Count Test Result

	blink count (times)
Control	7.80±1.75
Alkali	1.84±0.58
t	-0.373
P	0.713
d	0.167
95%CI	-1.988,1.388
